# Effects of Dietary Nucleotides on Growth Performance, Antioxidant Capacity, Intestinal Morphology and Gut Microbiota of Swamp Eel (*Monopterus albus*)

**DOI:** 10.3390/ani16131936

**Published:** 2026-06-23

**Authors:** Yueyun Han, Zijing Yuan, Bo Liu, Tianhai Liu, Qiwen Zhang, Zhe Zhang, Fuxian Zhang, Hanwen Yuan

**Affiliations:** College of Animal Science and Technology, Yangtze University, Jingzhou 434023, China; hanyueyun42@163.com (Y.H.); 18557531033@163.com (Z.Y.); liu_bo2026@163.com (B.L.); 17671358225@163.com (T.L.); qiwenjoe@163.com (Q.Z.); 13797469264@163.com (Z.Z.)

**Keywords:** nucleotides, *Monopterus albus*, intestinal morphology, growth performance, gut microbiota, antioxidant capacity

## Abstract

This study evaluated how graded dietary nucleotide supplementation (0, 0.25, 0.5, 0.75, 1.0, and 2.0 g/kg) affects growth performance, antioxidant capacity, intestinal morphology, and gut microbiota in swamp eel (*Monopterus albus*) (initial body weight 10.07 ± 0.92 g). Three hundred sixty fish were randomly assigned to six diets, each in triplicate, for eight weeks. Compared with the control, nucleotide addition significantly increased final body weight, weight gain rate, and specific growth rate, and decreased feed conversion ratio (*p* < 0.05), with optimal results at 0.75 g/kg (HS3). Survival was 100% in all groups. Supplemented fish showed lower serum and intestinal malondialdehyde levels and higher superoxide dismutase and catalase activities (*p* < 0.05). Serum total protein, albumin, and triglycerides increased, whereas alanine aminotransferase, aspartate aminotransferase, and γ-glutamyl transpeptidase decreased (*p* < 0.05), pointing to improved hepatic and lipid metabolism. Intestinal trypsin, lipase, and amylase activities also rose markedly (*p* < 0.05), peaking in HS3. Histological examination revealed greater mucosal thickness and villus height (*p* < 0.05); in HS3, these values reached approximately 0.95 mm and 0.87 mm, respectively. Metagenomic analysis showed that 0.75–1.0 g/kg nucleotides increased alpha diversity and restructured the microbial community, enriching Bacteroidetes- and Prevotella-related taxa while reducing Proteobacteria, including Acinetobacter baumannii and Escherichia coli. LEfSe identified dose-specific discriminant taxa, and refined KEGG Level 3 pathway analysis predicted enhanced butyrate and propanoate biosynthesis, starch utilization, and purine/pyrimidine interconversion at moderate doses. Genus-level abundances of Prevotella and Bacteroides correlated inversely with serum oxidative and hepatic stress markers. Quadratic regression estimated the optimal dietary nucleotide level at 764 mg/kg (0.76 g/kg), consistent with the best-performing 0.75 g/kg group. Collectively, 0.75–0.76 g/kg dietary nucleotides optimize growth and intestinal health in M. albus through coordinated improvements in antioxidant status, digestive function, mucosal architecture, and beneficial gut microbiota remodeling.

## Simple Summary

Swamp eels are an important farmed fish in China, but intensive farming practices and heavy antibiotic use have created problems with disease, poor growth, and environmental damage. This study investigated whether adding nucleotides—small building blocks of genetic material that are naturally found in living organisms—to the fish feed could improve their health and growth without relying on antibiotics. We fed groups of swamp eels with diets containing different amounts of nucleotides for eight weeks and measured their growth, health markers, intestinal structure, and gut bacteria. The results showed that adding nucleotides at a level of 0.75 g per kilogram of feed significantly improved the fish’s growth rate and feed efficiency, while also boosting their antioxidant defenses, digestive enzyme activity, and intestinal health. This treatment also increased the diversity of beneficial gut bacteria and reduced harmful bacteria. These findings suggest that dietary nucleotides can serve as a safe and effective alternative to antibiotics in swamp eel farming, supporting healthier fish and more sustainable aquaculture practices.

## 1. Introduction

The swamp eel (*Monopterus albus*) was first described and named in 1793 by Russian ichthyologist Basilius Zuiew. Traditionally, it has been considered widely distributed across Asia [1]. Among China’s paramount freshwater aquaculture varieties, it is primarily farmed in the southeastern and central regions, including the Yangtze River basin. China’s annual swamp eel production reached 355,000 tonnes in 2023, with Hubei, Jiangxi, Anhui, Hunan, and Sichuan provinces accounting for 96.4% of national output [2]. Currently, the industry focuses on high yield and efficiency, with production dominated by high-density intensive culture systems. While these practices have significantly increased output, they have also introduced challenges such as disease outbreaks, genetic bottlenecks, and environmental issues [3,4,5]. Additionally, intensive aquaculture, combined with severe pollution of aquatic environments, has led to an increase in disease occurrences, resulting in substantial economic losses for the industry [6]. At present, the prevention and control of aquatic diseases still rely heavily on antibiotics, leading to widespread antibiotic residues in water, sediments, and organisms within swamp eel farming systems [7]. Quinolones and sulfonamides are the predominant classes detected, with residue concentrations in pond water ranging from 0.6 to 92.2 ng/L and in sediments from 0.4 to 1169.3 ng/g dry weight [7]. High levels of antibiotics in the culture area can negatively impact the health of the swamp eel, causing stunted growth and compromising its immune system. This presents a significant risk to the industry’s sustainable development [8]. Consequently, there’s a pressing requirement to seek out potent substitutes for antibiotics, which can bolster disease resistance and foster sustainable, robust swamp eel farming.

For decades, feed additives have been widely used to prevent and reduce inflammation and related diseases in aquatic animals. These supplements enhance disease resistance and immune capabilities, potentially boosting growth and reproductive success [9,10]. For example, dietary supplementation with 3 g/kg of chitosan significantly enhanced antioxidant capacity and immune responses in hybrid sturgeon, reducing their susceptibility to bacterial infection [11]. Probiotic-based additives have also been reported to maintain immune competence in Nile tilapia fed low-protein diets and mitigate damage caused by ammonia-nitrogen stress [12]. Additionally, plant extracts containing terpenoids and organosulfur compounds exhibit antibacterial, anti-inflammatory, and immunomodulatory activities, contributing to improved antioxidant capacity and enhanced disease resistance [13].

Nucleotides are synthesized endogenously via de novo pathways, yet this process is energetically expensive and may not satisfy demand during rapid cell proliferation. Swamp eels under intensive culture exhibit high growth rates and rapid intestinal epithelial turnover. Under such conditions, dietary nucleotides become conditionally essential, sparing amino acids and energy for somatic growth rather than nucleic acid synthesis. Nucleotides, the fundamental precursors of nucleic acids, are essential building blocks of genetic material, including purine and pyrimidine nucleotides. These molecules are found throughout organs, tissues, and cells, playing critical roles in the transcription and translation of genetic information, regulating energy metabolism, and cell signaling [14]. It has been reported that supplementing flounder diets with 0.35% yeast-derived nucleotides can completely replace antibiotics and significantly improve weight gain and feed utilization. These perks stem from improved antioxidant power, a fact demonstrated by heightened functions of Cu-Zn SOD and GPx, and a boost in liver vitamin E concentrations. These factors ultimately help decrease mortality when faced with pathogens [15]. In juvenile golden pompano, dietary supplementation with 5′-inosine monophosphate greatly improved tolerance to salinity and oxidative stress while optimizing growth performance [16].

The application of nucleotides in aquatic animals has been widely studied. Research in Pacific white shrimp, grouper, and rainbow trout shows that dietary nucleotides can enhance immune function and improve growth performance [17,18,19]. However, evidence in swamp eel remains limited. To our knowledge, dose–response studies of dietary nucleotides in *M. albus* have not been reported; the only available study examined a single inclusion level (0.35%) in low-fishmeal diets of Japanese seabass (*Lateolabrax japonicus*) [20]. In the present study, we evaluated the effects of graded dietary nucleotide supplementation on growth performance, antioxidant capacity, intestinal morphology, and gut microbiota in *Monopterus albus*.

## 2. Materials and Experimental Methods

### 2.1. Material Source

The nucleotide supplement used in this study was a custom-formulated mixture of four 5′-monophosphate nucleotides: adenosine 5′-monophosphate (AMP), guanosine 5′-monophosphate (GMP), uridine 5′-monophosphate (UMP), and cytidine 5′-monophosphate (CMP). All four nucleotides were produced by enzymatic synthesis (purity ≥ 99% for each monomer) and obtained as disodium salts from Hubei Kaicheng Biotechnology Co., Ltd. (Yichang, China). The four monomers were mixed at an equal mass ratio (1:1:1:1) to prepare the final nucleotide supplement. No additional carrier or excipient was added. The supplement was stored at 4 °C in a desiccator prior to diet preparation. To ensure pristine quality, healthy swamp eels, scientifically known as *Monopterus albus*, were procured from the prestigious Aquaculture Breeding Base at Yangtze University.

### 2.2. Experimental Feed Formulation

The basal diet was formulated to meet the nutrient requirements for swamp eel growth (Table 1). Fish meal and soybean meal were the primary protein sources, soybean oil and soybean lecithin were the principal lipid sources, and wheat flour was the primary carbohydrate source. Nucleotides were supplemented at the expense of wheat flour to maintain isonitrogenous and isolipidic conditions across all treatments. Given the low inclusion levels (0.25–2.0 g/kg, ≤0.2% of diet), the contribution of the nucleotide supplement to total dietary protein, lipid, ash, and moisture was negligible; therefore, the proximate composition of the experimental diets was considered equivalent to that of the basal diet.

### 2.3. Experimental Design and Management

All feeding trials were carried out at the aquaculture facilities of Yangtze University. The culture system utilized fully aerated pond water and was exposed to natural sunlight. Before the experiment, swamp eels (*Monopterus albus*) were temporarily housed in greenhouse tanks and acclimated for one week. Three concrete tanks were used as independent culture units. Within each tank, six net cages (100 cm × 75 cm × 50 cm) were suspended in a circulating water system. The six dietary treatments (HS0–HS5) were randomly assigned to the six cages within each tank, with each treatment represented once per tank. Three hundred sixty healthy fish (initial body weight 10.07 ± 0.92 g) were randomly allocated to the 18 net cages, with 20 fish per cage. Each treatment therefore comprised three replicate cages (one per tank), with tank serving as a spatial block to ensure environmental replication.

The control group received a basal diet without nucleotide supplementation (0 mg/kg, HS0). The other five groups received supplemented diets at levels of 0.25 g/kg (HS1), 0.5 g/kg (HS2), 0.75 g/kg (HS3), 1.0 g/kg (HS4), and 2.0 g/kg (HS5). Dose levels were selected to bracket the anticipated optimum based on published effective ranges of dietary nucleotides in teleosts (typically 0.25–1.0 g/kg); the 2.0 g/kg level was included to evaluate potential adverse effects at excessive supplementation.

The eight-week feeding trial duration was sufficient for growth responses to manifest. Water hyacinth and other aquatic plants were added to each net cage to provide shelter. Fish were fed once daily at 18:00 at 2–5% body weight. Prepared feed dough was placed on floating nets to facilitate feeding. Uneaten feed was removed daily by siphoning 1 h after feeding. To avoid interference from water hyacinth roots, plants were gently shifted aside before siphoning. Residual pellets on the cage bottom were collected through a fine-mesh screen (0.5 mm). Any feed trapped in plant roots was dislodged by gentle shaking and rinsing with pond water. Collected residues were dried at 60 °C to constant weight and subtracted from the daily ration to calculate actual feed intake. Feeding quantities were adjusted daily based on visual assessment of feed consumption to maintain the target ration of 2–5% body weight.

Water depth was maintained at approximately 30 cm, with two-thirds of the water volume replaced every three days. Uneaten feed and excrement were removed daily. Eating behavior and water temperature were monitored during the experiment. Both the acclimation and experimental phases were conducted under consistent rearing conditions. Water temperature varied from 25 to 32 °C, dissolved oxygen averaged 4.0 ± 0.3 mg/L, pH levels ranged from 6.5 to 7.6, ammonia-nitrogen concentration was 0.060 ± 0.002 mg/L, and nitrite content was 0.040 ± 0.005 mg/L.

### 2.4. Sample Collection

At the end of the feeding trial, fish were fasted for 24 h before sampling. All surviving fish in each cage were counted to determine survival rate (SR). Thereafter, the 20 fish in each cage were individually weighed and measured for total length, and then dissected to obtain liver and viscera for hepatosomatic index (HSI) and viscerosomatic index (VSI) determination. Cage means were calculated for final body weight (FBW), weight gain rate (WGR), specific growth rate (SGR), condition factor (CF), HSI, and VSI. Feed conversion ratio (FCR) was computed per cage as total dry feed intake divided by total biomass gain (the difference between final and initial total body weight of all 20 fish in that cage).

For biochemical, histological, and microbiota analyses, five fish were randomly selected from the above 20 fish in each cage (15 fish per treatment). These fish were anaesthetized with MS-222 (Sigma-Aldrich, St. Louis, MO, USA). Blood was collected within 3 min of sedation to minimize stress-induced artefacts. Samples were kept at ambient temperature during collection, then allowed to clot at 37 °C for 1 h, followed by centrifugation at 4000× *g* for 15 min. Serum was stored at −20 °C until analysis. Intestinal tissues were isolated for digestive enzyme assays, antioxidant measurements, and histological analysis. Intestinal contents from the five fish of the same cage were pooled as one composite sample for gut microbiota analysis, yielding three samples per treatment.

### 2.5. Growth Performance

The following formulas were used to calculate the growth performance parameters:

Survival rate (SR, %) = 100% × (Final number of fish)/(Initial number of fish),

Weight gain rate (WGR, %) = 100% × (Final body weight (g) − Initial body weight (g))/(Initial body weight (g)),

Specific growth rate (SGR, %/day) = 100% × (Ln (Final body weight) − Ln (Initial body weight))/(Experimental duration (days)),

Condition factor (CF, g/cm^3^) = 100% × (Body weight (g))/(Body length (cm)^3^),

Viscerosomatic index (VSI, %) = 100% × (Viscera weight (g))/(Whole body weight (g)),

Hepatosomatic index (HSI, %) = 100% × (Liver weight (g))/(Whole body weight (g)).

### 2.6. Determination of Digestive Enzyme Activities, Serum Biochemical Parameters and Antioxidant Indices

Intestinal tissues were homogenized in pre-chilled physiological saline at a weight-to-volume ratio of 1:9. The homogenates underwent ultrasonic disruption and were then centrifuged at 4000× *g* for 10 min at 4 °C. The supernatants were collected for analysis. Intestinal digestive enzyme activities (amylase, trypsin, and lipase) were determined using colorimetric methods. Antioxidant-related parameters in the intestine and serum, including superoxide dismutase (SOD), malondialdehyde (MDA), and catalase (CAT), along with serum biochemical markers (total protein (TP), albumin (ALB), glucose (GLU), total cholesterol (TC), and triglycerides (TG)), were assessed using commercial diagnostic kits (Nanjing Bioengineering Institute, Nanjing, China) according to the manufacturer’s instructions. All assays were performed on an automated biochemical analyzer.

### 2.7. Intestinal Morphology

Foregut tissues were freshly collected, fixed in 4% paraformaldehyde, dehydrated through a graded ethanol series, cleared in xylene, and embedded in paraffin. Five-micrometer sections were then prepared and stained with hematoxylin and eosin (H&E). Images were captured using an Eclipse Ci-L light microscope (Nikon Corporation, Tokyo, Japan) at 20× magnification with consistent illumination. Morphometric analysis was conducted using Image-Pro Plus 6.0 software. Villus height and intestinal mucosal thickness were measured at five randomly selected locations per section, and the mean value was used for statistical analysis.

### 2.8. Intestinal Microbiota Composition and Diversity Analysis

Metagenomic sequencing of intestinal content samples (three replicates per treatment, 18 samples in total) was conducted by OE Biotech Co., Ltd. (Shanghai, China). Total microbial DNA was extracted using a commercial kit (Shanghai, China), and sequencing libraries were prepared with the TruSeq Nano DNA LT Sample Preparation Kit (Illumina, San Diego, CA, USA) and sequenced on an Illumina platform with paired-end reads. After quality filtering and removal of host-derived sequences, high-quality reads were obtained for downstream analyses.

De novo assembly and gene prediction were performed to generate a non-redundant gene set, which was taxonomically annotated against the NR database. Functional annotation was carried out by aligning the non-redundant gene set to the KEGG database to obtain KEGG Orthology (KO) information, and functional pathways were classified at KEGG Level B and Level C. Pathway abundances were expressed as relative abundances.

The microbial community’s composition was analyzed at the phylum, genus, and species levels. Alpha diversity was measured using the Shannon and Simpson indices, and beta diversity was analyzed by principal coordinate analysis (PCoA) utilizing Bray–Curtis distances. Taxa that exhibited differential abundance across treatments were detected by LEfSe with a linear discriminant analysis (LDA) score threshold of 3.0. An LDA threshold of 3.0 was chosen over the more commonly applied 2.0 to identify robust biomarkers and reduce false positives associated with the small sample size (n = 3 replicates per group); this more stringent criterion ensures that only taxa with strong effect sizes are reported as discriminant. All statistical analyses were conducted using R software, with significance established at *p* < 0.05.

### 2.9. Statistical Analysis

Each net cage served as the experimental unit for growth performance, digestive enzyme activity, antioxidant indices, serum biochemical parameters, and intestinal morphological measurements (n = 3 per treatment). Individual fish measurements within each cage were averaged to obtain cage means. Although cages were distributed across three concrete tanks to ensure spatial replication, preliminary analysis detected no significant tank effect on any measured parameter (*p* > 0.05); therefore, cage means were pooled across tanks and analyzed by one-way ANOVA followed by Tukey’s multiple comparisons test for pairwise comparisons using GraphPad Prism (version 9.5.0; GraphPad Software, San Diego, CA, USA). Significance was set at *p* < 0.05. For intestinal microbiota data, alpha diversity indices were compared by one-way ANOVA, beta diversity was assessed by principal coordinate analysis (PCoA) based on Bray–Curtis distances followed by analysis of similarities (ANOSIM), and linear discriminant analysis effect size (LEfSe) was performed with an LDA score threshold of 3.0 using R software (version 4.3.2; R Core Team, Vienna, Austria).

## 3. Results

### 3.1. Growth Performance

Table 2 shows the impact of varying levels of dietary nucleotide supplementation on the growth performance of *Monopterus albus.* No significant variations in beginning body weight were seen among the treatments (*p* > 0.05). Compared with the control group (HS0), dietary nucleotide supplementation significantly increased final body weight (FBW), weight gain rate (WGR) and specific growth rate (SGR), while significantly reducing feed conversion ratio (FCR) (*p* < 0.05). Among all treatments, fish in the HS3 group exhibited the best overall growth performance, with FBW, WGR and SGR increased by 39.5%, 61.6% and 28.0%, respectively, and FCR decreased by 37.8% compared with the HS0 group. Furthermore, FBW, WGR, and SGR in the HS2 group were markedly elevated compared to those in the HS0 group (*p* < 0.05), showing a similar trend to that observed in the HS3 group. Survival rate was 100% across all treatments, with no significant differences among groups (*p* > 0.05).

**Figure 1:**
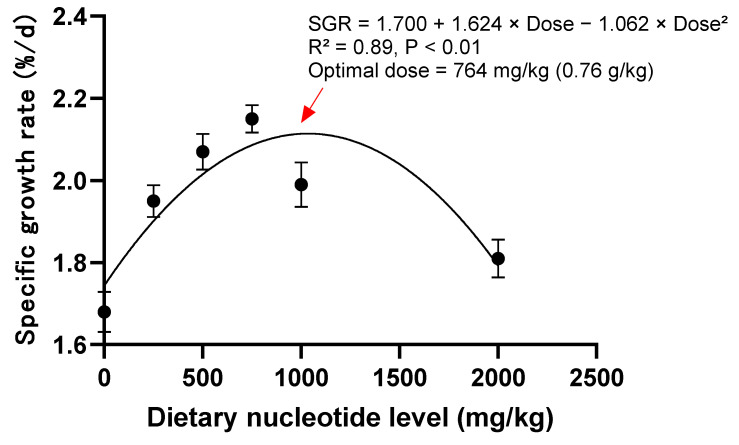
Quadratic regression analysis of specific growth rate (SGR) of swamp eel (*Monopterus albus*) in response to dietary nucleotide supplementation levels. The optimal dietary nucleotide level for maximum SGR was estimated to be 764 mg/kg (0.76 g/kg) based on the quadratic regression model (R^2^ = 0.89, *p* < 0.01).

The dose–response relationship between dietary nucleotide level and SGR was analyzed using quadratic regression (Figure 1). The fitted equation was: SGR = 1.700 + 1.624 × Dose − 1.062 × Dose^2^, which exhibited high fitting accuracy (R^2^ = 0.89, *p* < 0.01). The optimal dietary nucleotide inclusion level for maximizing SGR was calculated as 764 mg/kg (0.76 g/kg), consistent with the best growth performance recorded in the 0.75 g/kg HS3 group.

### 3.2. Antioxidant Capacity

#### 3.2.1. Serum Antioxidant Indices

**Figure 2:**
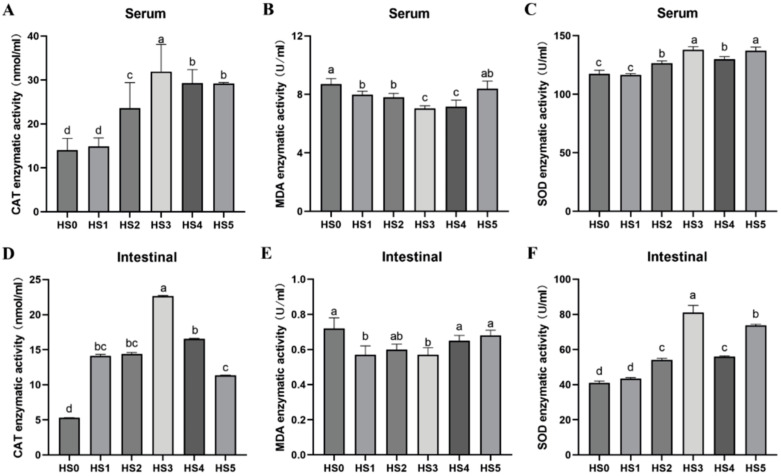
Serum and intestine antioxidant metrics in *Monopterus albus* following an 8-week feeding intervention with differing doses of dietary nucleotide supplementation. (**A**) Catalase (CAT) activity in serum; (**B**) malondialdehyde (MDA) content in serum; (**C**) superoxide dismutase (SOD) activity in serum; (**D**) intestinal CAT activity; (**E**) intestinal MDA content; (**F**) intestinal SOD activity. Data are expressed as mean ± SD (n = 3). Different lowercase letters above the columns denote significant differences among treatments (*p* < 0.05).

Figure 2A–C illustrate serum antioxidant indices. In comparison with the control group (HS0), dietary nucleotide supplementation considerably decreased serum malondialdehyde (MDA) levels and increased superoxide dismutase (SOD) and catalase (CAT) activities (*p* < 0.05). In particular, the HS3 group exhibited a lower MDA level (7.04 nmol/mL) and higher SOD (137.96 U/mL) and CAT activities (31.91 U/mL) than the HS0 group.

#### 3.2.2. Intestinal Antioxidant Indices

Intestinal antioxidant properties are illustrated in Figure 2D–F. The HS3 group demonstrated markedly elevated intestinal SOD and CAT activity in relation to the HS0 group (*p* < 0.05), with SOD increasing from 41.07 to 81.02 U/mL and CAT attaining 22.65 U/mL. Intestinal MDA concentrations were typically decreased in the nucleotide-supplemented cohorts compared to the control group.

### 3.3. Serum Biochemical Indices

The serum biochemical parameters are summarized in Table 3. Liver-related indices differed significantly among treatments (*p* < 0.05). Compared with the control (HS0), enzyme activity of alanine aminotransferase (ALT) and aspartate aminotransferase (AST) exhibited a downward trend in the HS3–HS5 groups, with the greatest changes observed in fish fed the HS3 diet. In contrast, γ-glutamyl transpeptidase (γ-GT) activity was consistently lower in the nucleotide-supplemented groups and reached the lowest value in the HS3 group (*p* < 0.05). With respect to protein metabolism, fish in the HS2 and HS3 groups showed higher serum total protein (TP) and albumin (ALB) levels than those in the control group (*p* < 0.05). For carbohydrate and lipid metabolism, serum glucose (GLU) and total cholesterol (CHO) were not significantly affected by dietary treatments, whereas triglyceride (TG) levels were significantly elevated in the HS2 and HS3 (*p* < 0.05).

### 3.4. Digestive Enzyme Activities

**Figure 3:**
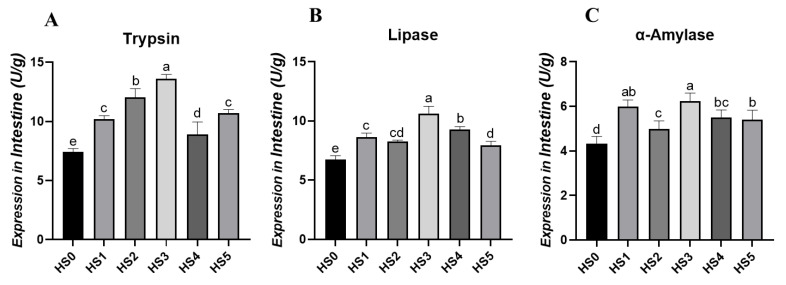
Intestinal digestive enzyme activities of *Monopterus albus* following an 8-week feeding trial with different dietary nucleotide supplementations. (**A**) Trypsin activity; (**B**) Lipase activity; (**C**) α-amylase activity. Data are expressed as mean ± SD (n = 3). Significant differences between treatments are denoted by different lowercase superscript letters (*p* < 0.05).

The effects of dietary nucleotides on intestinal digestive enzyme activities of Monopterus albus are shown in Figure 3. Fish fed nucleotide-supplemented diets exhibited markedly elevated intestinal trypsin, lipase, and α-amylase activities compared to the control group (*p* < 0.05). In the HS3 group, trypsin activity reached 13.50 ± 0.40 U/g, lipase activity reached 10.64 ± 0.61 U/g, and α-amylase activity reached 6.24 ± 0.37 U/g, representing increases of approximately 81%, 58%, and 44% over the HS0 control (7.44 ± 0.26, 6.74 ± 0.34, and 4.33 ± 0.33 U/g, respectively). Activity peaked in the HS3 group for all three enzymes, which differed significantly from the HS0 group (*p* < 0.05). The HS2 and HS5 groups showed moderate increases compared to the control (*p* < 0.05).

### 3.5. Intestinal Morphology

**Figure 4:**
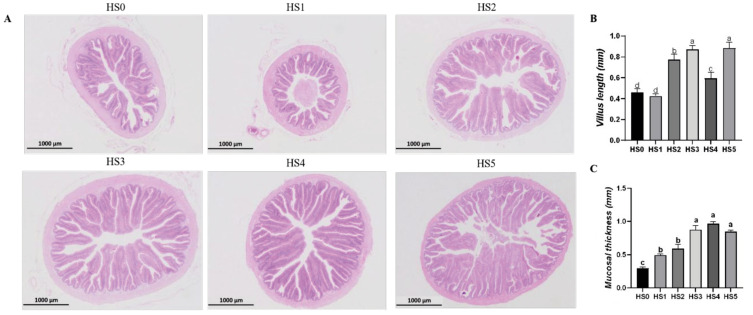
The impact of dietary nucleotide supplementation on the front portion of the intestine in *Monopterus albus*. (**A**) Representative histological photographs of the anterior intestine; (**B**) Villus height; (**C**) Intestinal mucosal thickness. Scale bar = 1000 μm. Data are expressed as mean ± standard deviation (n = 3). Distinct superscript letters indicate significant differences between treatment groups (*p* < 0.05).

Figure 4A shows representative intestinal histology sections. Compared with the control group (HS0), dietary nucleotide supplementation noticeably altered intestinal tissue morphology. Fish in the HS3–HS5 groups displayed a more intact transverse intestinal structure with a fuller tissue appearance. As illustrated in Figure 4C, intestinal mucosal thickness was significantly increased in the nucleotide-supplemented groups (*p* < 0.05). The highest values were observed in the HS3 and HS4 groups, reaching approximately 0.95–1.00 mm (950–1000 μm), which were markedly higher than that of the HS0 group (about 0.30 mm, i.e., 300 μm). The HS2 and HS5 groups also demonstrated significantly increased mucosal thickness compared to the control group (*p* < 0.05). Quantitative examination of villus morphology (Figure 4B) demonstrated a notable augmentation in villus length in the HS2, HS3, and HS5 groups relative to the HS0 group (*p* < 0.05). Villus length in the HS3 and HS5 groups reached approximately 0.87 mm and 0.88 mm, respectively, whereas the HS4 group exhibited an increase to about 0.77 mm. No substantial difference was observed between the HS1 and HS0 groups (*p* > 0.05), and the increase in the HS2 group was inferior to that in the HS3 and HS5 groups.

### 3.6. Effects of Dietary Nucleotides on Intestinal Microbial Community Structure, Composition and Differential Taxa

#### 3.6.1. Effects of Dietary Nucleotides on Intestinal Microbial Diversity

**Figure 5:**
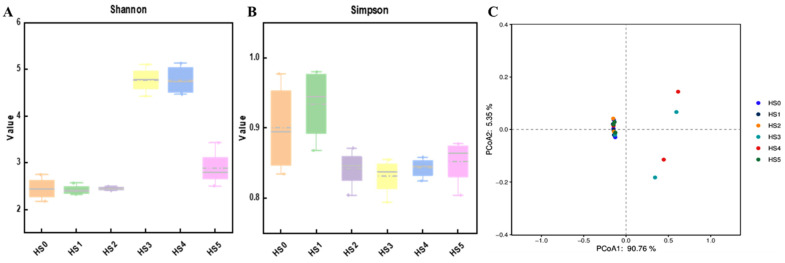
The impact of dietary nucleotide supplementation on the alpha and beta diversity of the intestinal microbiota in *Monopterus albus*. (**A**,**B**): boxplots of the Shannon and Simpson indices showing the median, interquartile range, and minimum–maximum values. (**C**): A distance matrix-based principal coordinate analysis (PCoA) was conducted, with the percentage of variation accounted for by each axis indicated in parentheses (HS0–HS5). ANOSIM analysis indicated a weak separation of microbial community structure among treatments, which did not reach statistical significance (R = 0.17, *p* = 0.051). n = 3.

Alpha diversity analysis showed that diets containing different levels of nucleotides significantly influenced the intestinal microbial diversity of *Monopterus albus* (Figure 5A,B). In comparison to the HS0 group, the Shannon index exhibited a considerable rise in the HS3 and HS4 groups, accompanied by a decrease in the Simpson index, indicating an enhancement in microbial diversity without a substantial alteration in community evenness. No significant variations in alpha diversity were detected among the other treatment groups (HS1, HS2, and HS5). Principal coordinate analysis (PCoA) (Figure 5C) was used to further assess the overall differences in the structure of intestinal microbial communities between treatments. The first principal coordinate (PCoA1) explained 90.76% of the total variation. Samples from the control and low-level nucleotide groups (HS0, HS1, HS2, and HS5) largely overlapped in the ordination space, indicating similar microbial community structures. In contrast, samples from the HS3 and HS4 groups were clearly separated along the horizontal axis, forming distinct clusters, which suggests that these levels of nucleotide supplementation markedly altered the intestinal microbial community structure compared with the other groups. However, ANOSIM analysis showed that the overall separation among treatments was not statistically significant.

#### 3.6.2. Effects of Dietary Nucleotides on the Taxonomic Composition of the Intestinal Microbiota

**Figure 6:**
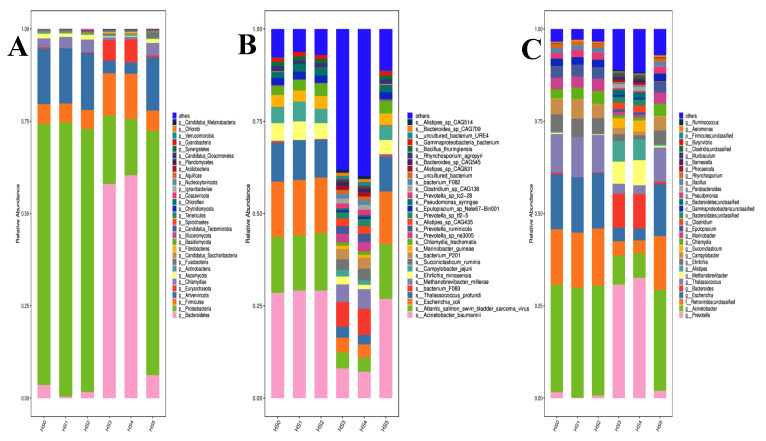
Relative abundance of the intestinal microbial composition in *Monopterus albus* under different dietary treatments. (**A**) Phylum level; (**B**) species level; (**C**) genus level. Only major taxa are shown, and the remaining taxa are grouped as “Others” (HS0–HS5). n = 3.

At the phylum level (Figure 6A), the intestinal microbiota across all treatment groups was predominantly composed of Proteobacteria, Firmicutes, and Bacteroidetes. ANOVA confirmed that Bacteroidetes and Firmicutes relative abundances were significantly higher in the HS3 and HS4 groups than in the HS0, HS1, and HS2 groups (*p* < 0.05), whereas Proteobacteria was significantly reduced in the HS4 group compared to the control (*p* < 0.05). In the HS5 group, alterations in phylum-level composition were noted, with Bacteroidetes and Firmicutes at intermediate levels and the overall microbial structure exhibiting greater similarity to that of the low-dose groups.

At the genus level (Figure 6B), Acinetobacter, Escherichia, and Bacteroides were the principal taxa in the control and some treatment groups (HS0, HS1, HS2, and HS5). In the medium-dose groups (HS3 and HS4), a noticeable change in community composition was observed, with a significant increase in *Prevotella*, while the relative abundances of Escherichia and Acinetobacter were significantly reduced. In the HS5 group, there was a reduction in *Prevotella*, while Acinetobacter and Escherichia levels increased again.

At the species level (Figure 6C), *Acinetobacter baumannii* and *Escherichia coli* were the dominant species in the HS0–HS2 groups, with the highest relative abundance of *E. coli* detected in the HS2 group. In the HS3 and HS4 groups, the proportions of these dominant species were substantially reduced, whereas several *Prevotella*-related species (including *Prevotella ruminicola* and *Prevotella* sp.) were notably increased. The HS5 group saw a renewed increase in *A. baumannii* and *E. coli*, leading to a microbial profile that contrasted sharply with the profiles of the HS3 and HS4 groups.

#### 3.6.3. Effects of Dietary Nucleotides on Differential Intestinal Microbial Taxa

**Figure 7:**
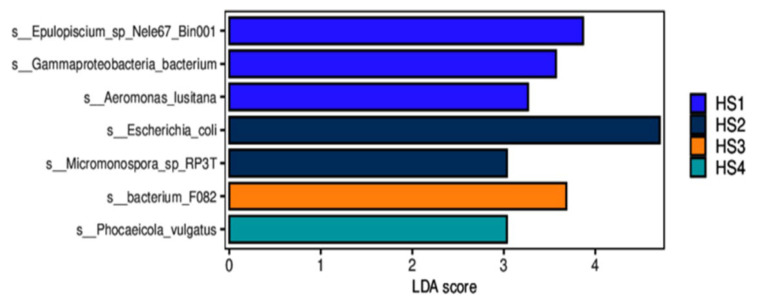
LEfSe analysis of differential microbial biomarkers in the intestinal microbiota of *Monopterus albus* among different dietary treatments. The length of each bar represents the Linear Discriminant Analysis (LDA) score, reflecting the effect size, while the colors indicate taxa that are enriched in the respective treatment groups (HS1–HS4). Sample size: n = 3.

To identify indicator taxa contributing to microbial differences among dietary nucleotide treatments, linear discriminant analysis effect size (LEfSe) was performed (Figure 7). The results showed that each treatment group was characterized by distinct taxa with relatively high LDA scores, indicating that dietary nucleotide supplementation influenced the intestinal microbial community at the species level. In the HS1 group, *Epulopiscium* sp. Nele67-Bin001, *Gammaproteobacteria* bacterium, and Aeromonas lusitana were identified as the predominant differential taxa. The HS2 group was mainly characterized by *Escherichia coli* and *Micromonospora* sp. RP3T. In contrast, in the medium-dose nucleotide groups, bacterium F082 was significantly enriched in the HS3 group, whereas *Phocaeicola vulgatus* was identified as the most representative differential taxon in the HS4 group, exhibiting the highest LDA score. Overall, the LEfSe results were consistent with the taxonomic composition analysis, further indicating that moderate dietary nucleotide supplementation was associated with distinct shifts in the intestinal microbial community structure.

#### 3.6.4. Effects of Dietary Nucleotides on Functional Characteristics of the Intestinal Microbiota

**Figure 8:**
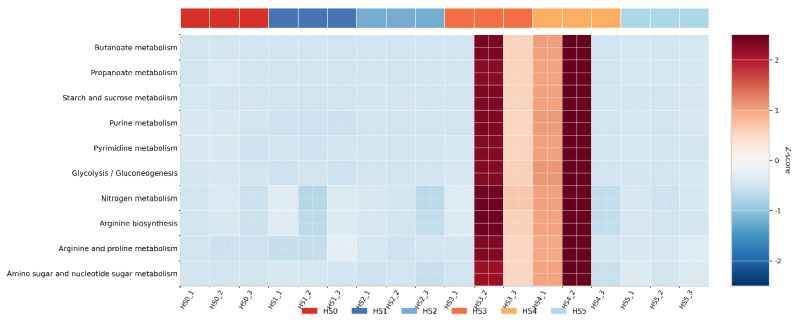
Heatmap of selected KEGG Level C pathways in the intestinal microbiota of *Monopterus albus* (row z-score normalized). Samples (columns) and pathways (rows) were clustered using Pearson correlation distance with average linkage. The 0.75 g/kg (HS3) and 1.0 g/kg (HS4) groups formed a distinct cluster from the control (HS0) and low-dose treatments (HS1, HS2), exhibiting significantly higher predicted abundances in starch and sucrose metabolism (ko00500; *p* < 0.05), butanoate metabolism (ko00650; *p* < 0.05), and propanoate metabolism (ko00640; *p* < 0.05), along with elevated purine metabolism (ko00230) and pyrimidine metabolism (ko00240). The 2.0 g/kg group (HS5) clustered with suboptimal doses, suggesting a dosage ceiling beyond which further functional enhancement is not achieved. Functional annotation was performed against the KEGG database using metagenomic sequencing data.

We first examined KEGG Level 2 functional profiles and found that broad metabolic categories were comparable across all six dietary groups, as is typical for metagenomic functional profiling. However, refined analysis at the Level 3 pathway level revealed dose-dependent functional signatures. Hierarchical clustering of selected pathways involved in energy acquisition, polysaccharide degradation, and nucleotide catabolism clearly separated the 0.75 g/kg (HS3) and 1.0 g/kg (HS4) groups from the control (0 g/kg, HS0) and low-dose groups (0.25 g/kg, HS1; 0.5 g/kg, HS2) (Figure 8). ANOVA confirmed that starch and sucrose metabolism (ko00500) was significantly enriched in the HS3 and HS4 groups compared to the control and low-dose groups (*p* < 0.05). Butanoate metabolism (ko00650) was significantly elevated in the HS4 group relative to the control (*p* < 0.05), whereas propanoate metabolism (ko00640) showed a significant increase in the HS4 group compared to HS1 and HS2 (*p* < 0.05). Moderate nucleotide supplementation therefore enhanced predicted capacities for butanoate and propanoate biosynthesis—two major short-chain fatty acid (SCFA) production pathways—along with increased potential for starch and sucrose utilization and purine/pyrimidine interconversion. These findings indicate that dietary nucleotides at 0.75–1.0 g/kg selectively promote the ability of specific intestinal microbial guilds to degrade complex carbohydrates and utilize exogenous nucleotides, thereby augmenting overall fermentative activity of the intestinal ecosystem. In contrast, the 2.0 g/kg group (HS5) failed to maintain these beneficial functional changes for starch metabolism, implying a dosage threshold above which additional nucleotide supplementation yields no further metabolic improvements.

#### 3.6.5. Association Between Differentially Abundant Taxa and Host Phenotypic Indices

**Figure 9:**
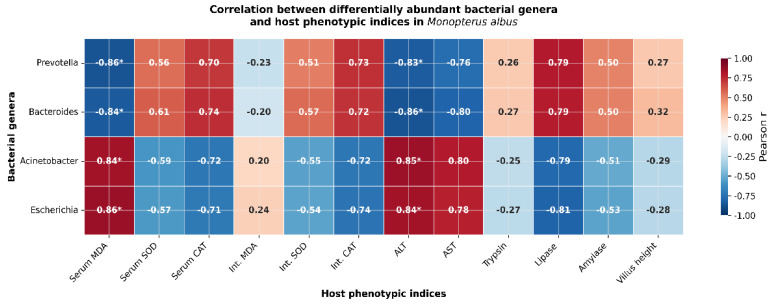
Pearson correlation heatmap between differentially abundant bacterial genera and host phenotypic indices in Monopterus albus. Correlation coefficients (r) were calculated from treatment means (n = 6) and are color-coded (red, positive; blue, negative). * *p* < 0.05 (two-tailed). Prevotella and Bacteroides displayed inverse relationships with serum MDA and ALT, and positive correlations with lipase activity (*p* = 0.061), whereas Acinetobacter and Escherichia showed the opposite pattern. These associations are consistent with the metabolic capacities of these taxa but do not establish causality.

To determine whether the altered microbiota composition was associated with host phenotypic improvements, we performed Pearson correlation analysis between genus-level relative abundances and key physiological indices across the six treatments. *Prevotella* and *Bacteroides*, which were more abundant in the 0.75 g/kg and 1.0 g/kg groups, displayed strong inverse relationships with serum MDA (r = −0.86 and −0.84, respectively, *p* < 0.05) and ALT (r = −0.83 and −0.86, respectively, *p* < 0.05), and positive correlations with lipase activity (r = 0.79 for both, *p* = 0.061) (Figure 9). In contrast, Acinetobacter and Escherichia, which were suppressed at these doses, exhibited the opposite pattern: they correlated positively with serum MDA (r = 0.84 and 0.86, *p* < 0.05) and negatively with lipase activity (r = −0.79 and −0.81, *p* < 0.10). These statistical associations are consistent with the established metabolic capacities of these taxa: both *Prevotella* and *Bacteroides* ferment polysaccharides to produce SCFAs, whereas Acinetobacter and Escherichia (members of Gammaproteobacteria) are often linked to dysbiosis and pro-inflammatory lipopolysaccharide signaling. While these correlative associations do not imply causality, they provide a testable hypothesis that nucleotide-driven microbiota remodeling supports host intestinal and metabolic health by enhancing beneficial fermentation and reducing the abundance of potentially pathogenic taxa.

## 4. Discussion

Supplementing the diet with nucleotides markedly boosted final body weight, growth rate, and specific growth rate, while lowering the feed conversion ratio, suggesting enhanced feed efficiency in *Monopterus albus*. This finding agrees with the growth-promoting effects of nucleotides reported in other aquatic species, including turbot, tilapia and grass carp [21,22,23]. As conditionally essential nutrients, nucleotides can conserve energy that would otherwise go into de novo synthesis during periods of fast growth or stress, freeing up resources for tissue growth and repair [24]. Quadratic regression analysis of SGR further quantified the dose–response relationship and estimated the optimal dietary nucleotide level at 764 mg/kg (0.76 g/kg), which closely matched the optimal growth performance observed in the 0.75 g/kg HS3 group. This consistency confirms that appropriate nucleotide inclusion can maximize growth potential, whereas excessive supplementation (≥1000 mg/kg) fails to produce additional growth benefits. The lack of further improvement at 2.0 g/kg (HS5) suggests a dose ceiling. Plausible mechanisms include saturation of nucleoside salvage pathways, leading to feedback inhibition of de novo purine synthesis and increased renal excretion of nitrogenous metabolites. Alternatively, excess nucleotides may alter hepatic lipogenic flux or increase the metabolic cost of nucleotide catabolism, diverting energy away from somatic growth. High concentrations of nucleotide metabolites such as uric acid may also exert osmotic stress. These hypotheses remain speculative and require metabolomic profiling to confirm. However, the high-dose group (HS5) did not show further improvements in growth performance, suggesting a dose-dependent response; excessive supplementation may reduce nutrient utilization efficiency or increase metabolic burden, as also reported in other fish studies [25]. Notably, the dose-dependent growth response paralleled the microbial community shifts (Figure 5, Figure 6 and Figure 7), suggesting that optimal nucleotide levels may promote growth partly through gut microbiota modulation.

Serum biochemical indices are important indicators of metabolic status and overall health. The current study found that nucleotide supplementation improved serum biochemical parameters, which was associated with a boost in antioxidant capacity, indicated by reduced MDA levels and elevated SOD and CAT activities. Previous studies have shown that nucleotides can strengthen antioxidant defence systems and reduce lipid peroxidation, thereby alleviating oxidative stress-related tissue damage [26,27]. The HS3 group exhibited the greatest improvement in antioxidant indices, suggesting that an appropriate nucleotide level helps maintain redox homeostasis and provides a favourable physiological basis for growth. The concurrent drop in serum ALT and AST across HS3–HS5 points to reduced hepatic stress, possibly reflecting both diminished oxidative damage and lower exposure to gut-derived bacterial products. Escherichia and Acinetobacter—both suppressed at 0.75–1.0 g/kg (Figure 6)—are known to produce lipopolysaccharide (LPS) and other pro-inflammatory compounds. Their decline in the moderate nucleotide groups may therefore lower the luminal burden of these hepatotoxic factors. The inverse tracking of *Prevotella* and *Bacteroides* with MDA and ALT (Figure 9) is compatible with this proposed gut–liver protective axis, although the specific molecular mediators (e.g., tight junction proteins, inflammatory cytokines) remain to be identified.

The intestine is a key organ for digestion and nutrient absorption, and its structural and functional status directly affects growth performance. Dietary nucleotides increased intestinal digestive enzyme activities, with the most pronounced effects in the HS3 group, consistent with the role of nucleotides in supporting intestinal epithelial renewal [28]. Histological observations showed that moderate nucleotide supplementation increased mucosal thickness and villus length, enlarging the absorptive surface area, as also reported in carp and turbot [29,30]. The improved intestinal morphology in HS3/HS4 coincided with enrichment of *Prevotella* and *Bacteroidetes* (Figure 6), which are specialized polysaccharide degraders. Metagenomic functional prediction revealed elevated butanoate and propanoate metabolism pathways in these groups (Figure 8), providing functional evidence consistent with enhanced microbial fermentation. While direct SCFA quantification was not performed, the co-occurrence of *Prevotella* enrichment, SCFA pathway upregulation, and improved intestinal architecture suggests a microbiota–fermentation–barrier axis as a plausible mechanism, awaiting validation through direct measurement of fecal SCFAs and intestinal tight junction proteins.

The intestinal microbiota of teleost fish acts as a metabolic interface that transforms dietary inputs into host-available signals [31]. In this study, the 0.75–1.0 g/kg nucleotide groups (HS3 and HS4) separated from the control along a clear directional trajectory: the PCoA first axis captured 90.76% of the variance, and the Shannon and Simpson indices followed patterns distinct from HS0 (Figure 5). This directional shift suggests a modest community reorganization, although the overall separation did not achieve strict statistical significance (ANOSIM, R = 0.17, *p* = 0.051). The phylum-level redistribution—specifically the Bacteroidetes and Firmicutes gain and Proteobacteria loss in HS3/HS4 (Figure 6A)—suggests a metabolic rewiring. Bacteroidetes carry an extensive repertoire of carbohydrate-active enzymes and polysaccharide-utilizing loci that break down starch and plant cell-wall polymers into fermentable sugars [32]. Our KEGG annotation picked up this signal directly: starch and sucrose metabolism (ko00500), butanoate biosynthesis (ko00650), and propanoate biosynthesis (ko00640) all climbed in HS3/HS4 relative to HS0–HS2 (Figure 8). These three pathways funnel into short-chain fatty acid (SCFA) output, especially butyrate and propionate. In mammals and, more recently, in several fish species, butyrate is recognized as the preferred fuel for colonocyte respiration; it is also reported to strengthen epithelial barriers, putatively by GPR41/43-mediated signaling and histone deacetylase inhibition, which may in turn support tight-junction protein expression (e.g., ZO-1, occludin) [2,33]. Propionate reaches the liver via the portal vein and has been shown to dampen hepatic gluconeogenesis and systemic inflammation in vertebrate models [34]. Whether these exact receptor pathways operate in swamp eel remains unknown, but the predicted pathway enrichment in our dataset is at least compatible with this literature framework. The enrichment of Prevotella-related taxa at the genus and species levels in HS3/HS4 (Figure 6), backed by the LEfSe biomarker analysis (Figure 7), fits this picture. *Prevotella* spp. are well-established polysaccharide degraders in vertebrate intestines and are among the dominant SCFA producers in aquatic omnivorous species [35]. At the same time, the drop in Acinetobacter and Escherichia—both Gammaproteobacteria frequently flagged as dysbiotic expanders [32]—is unlikely to be neutral. These taxa are common sources of lipopolysaccharide (LPS); their suppression should, in principle, reduce luminal endotoxin load and the downstream pro-inflammatory signaling that LPS typically triggers [36]. We did not measure LPS or inflammatory cytokines in this trial, so this link remains a working assumption. Beyond carbohydrate fermentation, the elevated purine (ko00230) and pyrimidine (ko00240) metabolism in HS3/HS4 points to another ecological advantage: the supplemented nucleotides likely served as exogenous substrates that selected for microbial guilds skilled at nucleoside salvage and interconversion. This microbial activity may feed back into host nucleotide pools, possibly easing the metabolic cost of de novo synthesis during the rapid growth phase documented in HS3 (Table 2) [24]. Curiously, the 2.0 g/kg group (HS5) lost these gains. The microbial profile slid back toward the control state, with Acinetobacter and Escherichia rebounding (Figure 6) and the SCFA pathways falling back to baseline (Figure 8). We interpret this as an ecological ceiling: at excessive nucleotide concentrations, the redox environment or competitive dynamics may shift in ways that no longer favor Bacteroidetes-dominated fermentation.

To test whether these microbial changes tracked with host physiology, we correlated genus-level abundances against phenotypic indices (Figure 9). Prevotella and Bacteroides in HS3/HS4 correlated negatively with serum MDA (r = −0.86 and −0.84, respectively, *p* < 0.05) and ALT (r = −0.83 and −0.86, respectively, *p* < 0.05), and positively with lipase activity (r = 0.79 for both, *p* = 0.061). Acinetobacter and Escherichia showed the mirror image: positive links to MDA (r = 0.84 and 0.86, *p* < 0.05) and negative links to lipase (r = −0.79 and −0.81, *p* < 0.10). The direction of these correlations makes biological sense. Prevotella and Bacteroides are polysaccharide fermenters whose end-products include SCFAs; in fish as in mammals, SCFAs are documented to support intestinal epithelial renewal and to stimulate digestive enzyme secretion, although the exact endocrine intermediates—such as GLP-2 or PYY—have not been characterized in *M. albus* [37,38]. The improved villus height and mucosal thickness in HS3/HS4 (Figure 4) may therefore partially reflect microbial fermentation signals rather than a direct pharmacological effect of dietary nucleotides alone. On the other side, the suppression of LPS-bearing *Acinetobacter* and *Escherichia* would be expected to relieve pressure on the TLR–MyD88–NF-κB axis, a canonical LPS-sensing pathway well described in mammalian gut–liver studies and increasingly reported in teleost immunity [39,40]. Lower endotoxin exposure would plausibly reduce hepatic inflammation and oxidative damage, matching the drop in ALT, AST, and γ-GT (Table 3) and the MDA decline. Again, we did not assay TLR4 expression, NF-κB activation, or luminal LPS directly; these molecular steps are inferred from the taxonomic and phenotypic patterns, not demonstrated experimentally herein. Collectively, the correlations, the predicted pathway enrichment, and the dose-specific community restructuring suggest that 0.75–1.0 g/kg dietary nucleotides acts as an ecological filter: they enrich carbohydrate-fermenting, SCFA-producing guilds and deplete potential dysbiotic Proteobacteria, creating an intestinal environment that coincides with reduced oxidative load and better digestive output. The hypothesis that a microbiota–gut–liver axis mediates these benefits is consistent with the data but remains to be tested. Direct measurement of fecal SCFAs, luminal LPS, intestinal tight-junction proteins (e.g., ZO-1, occludin), and key inflammatory markers (e.g., TLR4, IL-1β, TNF-α) should be prioritized in follow-up work to move from correlation to causation.

Taken together, the growth, physiological and biochemical responses, intestinal morphology, and microbial results suggest that dietary nucleotides promote growth in *Monopterus albus* through coordinated effects on antioxidant status, digestive capacity, intestinal structure and gut microbial community. Among the tested levels, the HS3 supplementation level produced the most favourable overall outcomes.

This study has several limitations. The open pond cage system meant that wild plankton and organic matter on water hyacinth roots could be ingested by fish but were not recorded as feed intake. This uncontrolled natural food source may have influenced absolute FCR values, although relative differences among treatments remain valid because all cages were exposed to the same environmental conditions. Second, ANOSIM analysis showed that overall microbial community separation among treatments was marginally non-significant (R = 0.17, *p* = 0.051). While specific taxa shifted and functional pathways differed, the global community structure did not reach statistical significance. These findings should therefore be interpreted as directional trends rather than robust community-level restructuring.

## 5. Conclusions

This study demonstrated that incremental dietary nucleotide supplementation up to 0.75–1.0 g/kg markedly improved growth performance, antioxidant capacity, serum protein and lipid profiles, digestive enzyme activities, and intestinal morphology of *Monopterus albus.* Metagenomic analysis further revealed that this dose range restructured the gut microbiota, enriching Bacteroidetes- and Prevotella-related taxa while suppressing Proteobacteria (Acinetobacter and Escherichia), and enhanced predicted capacities for butyrate/propionate biosynthesis and nucleotide salvage. These coordinated improvements suggest that moderate nucleotide supplementation may promote host growth and intestinal health, at least in part, through a microbiota–gut–liver axis involving enhanced microbial fermentation and reduced luminal pro-inflammatory burden; however, the proposed molecular links (e.g., SCFA receptor signaling, TLR4–NF-κB modulation) remain to be experimentally validated.

Quadratic regression analysis based on SGR estimated the optimal dietary nucleotide level at 764 mg/kg (0.76 g/kg), which closely matched the 0.75 g/kg treatment exhibiting the best overall performance. Therefore, a dietary nucleotide inclusion of 0.75–0.76 g/kg is recommended for practical feed formulation in swamp eel aquaculture.

## Figures and Tables

**Table 1:** Ingredient and proximate composition of the basal diet (on a dry matter basis).

Ingredients (%)	Composition
Fish meal	45
Soybean meal	12
Corn protein powder	5
Wheat flour	25
Compound protein ^1^	2
Shrimp meal	1.5
Soybean oil	2
Soybean lecithin	2
Premix ^2^	1
Earthworm meal	2
Dicalcium phosphate	2
Choline	0.3
Phytase	0.2
Proximate composition	
Moisture	12.37
Crude protein	44.78
Crude lipid	7.92
Ash	11.64

Gross energy was not determined analytically; however, diets were formulated to be isoenergetic by adjusting the wheat flour content to compensate for nucleotide supplementation. ^1^ Complex protein may suggest that soybean protein concentrate provides a significant foundation for its formulation. ^2^ The premix could indicate that the following nutrients were provided per kg of the diet: FeSO_4_⋅7H_2_O 150.0 mg; ZnSO_4_⋅H_2_O 50.0 mg; MgSO_4_⋅7H_2_O 500.0 mg; MnSO_4_⋅4H_2_O 20.0 mg; CuSO_4_⋅5H_2_O 10.0 mg; NaCl 1000.0 mg; KI 0.8 mg; CoCl_2_⋅6H_2_O 0.4 mg; NaSeO_3_⋅5H_2_O 0.3 mg; VD_3_ 2500.0 IU; VC 200.0 mg; VE 200.0 mg; VB_2_ 45.0 mg; nicotinic acid 200.0 mg; Ca-pantothenate 60.0 mg; inositol 200.0 mg; VB_1_ 25.0 mg; VB_6_ 20.0 mg; VK_3_ 10.0 mg; folic acid 10.0 mg; biotin 1.5 mg; VB_12_ 0.1 mg.

**Table 2:** Effects of dietary nucleotide supplementation on growth performance of *M. albus* after an 8-week feeding trial.

Item	Groups					
	(HS0)	(HS1)	(HS2)	(HS3)	(HS4)	(HS5)
IBW/g	10.07 ± 0.90	10.07 ± 0.89	10.07 ± 0.94	10.07 ± 0.92	10.07 ± 0.88	10.07 ± 0.91
FBW/g	26.69 ± 2.63 ^d^	32.31 ± 3.08 ^bc^	36.28 ± 4.69 ^a^	37.22 ± 3.05 ^a^	29.11 ± 2.87 ^c^	34.35 ± 5.36 ^ab^
WGR/%	162.13 ± 28.71 ^d^	230.6 ± 29.82 ^b^	248.46 ± 35.18 ^ab^	262.02 ± 30.13 ^a^	217.15 ± 35.04 ^c^	233.45 ± 44.19 ^b^
SR/%	100 ± 0	100 ± 0	100 ± 0	100 ± 0	100 ± 0	100 ± 0
SGR/ (%/d)	1.68 ± 0.19 ^d^	1.95 ± 0.15 ^b^	2.07 ± 0.17 ^ab^	2.15 ± 0.13 ^a^	1.81 ± 0.18 ^c^	1.99 ± 0.21 ^b^
FCR	1.80 ± 0.41 ^a^	1.20 ± 0.27 ^bc^	1.07 ± 0.25 ^cd^	1.05 ± 0.18 ^d^	1.37 ± 0.28 ^b^	1.10 ± 0.36 ^cd^
HSI%	3.55 ± 0.6 ^d^	4.23 ± 0.71 ^b^	4.54 ± 0.91 ^b^	4.98 ± 1.04 ^a^	3.92 ± 0.67 ^c^	4.49 ± 1.57 ^b^
VSI%	10.09 ± 1.38 ^c^	10.8 ± 2.19 ^b^	11.45 ± 1.79 ^a^	11.9 ± 2.03 ^a^	10.76 ± 1.41 ^b^	10.81 ± 1.36 ^b^
CF/(g/cm^3^)	0.08 ± 0.01	0.09 ± 0.01	0.09 ± 0.01	0.09 ± 0.01	0.09 ± 0.01	0.08 ± 0.01

Within the same row, values marked with different lowercase superscripts (a–d) indicate significant differences between treatments (*p* < 0.05). Each treatment was conducted in triplicate, and results are presented as mean ± standard deviation (SD). Abbreviations: IBW, initial body weight; FBW, final body weight; SR, survival rate; WGR, weight gain rate; SGR, specific growth rate; FCR, feed conversion ratio; HSI, hepatosomatic index; VSI, viscerosomatic index; CF, condition factor.

**Table 3:** Serum biochemical indices of *M. albus* fed diets containing assessed tiers of nucleotides following an 8-week feeding trial.

Item	Groups					
	(HS0)	(HS1)	(HS2)	(HS3)	(HS4)	(HS5)
ALT(U/L)	5.19 ± 0.83 ^a^	5.07 ± 0.51 ^a^	4.64 ± 0.27 ^b^	3.36 ± 0.44 ^c^	3.47 ± 0.41 ^c^	3.82 ± 0.59 ^b^
AST(U/L)	132.81 ± 1.62 ^a^	127.46 ± 1.91 ^a^	126.4 ± 0.14 ^a^	103.09 ± 2.25 ^b^	104.54 ± 1.32 ^b^	105.64 ± 1.59 ^b^
γ-GT(U/L)	1.3 ± 0.17 ^a^	1.13 ± 0.02 ^ab^	0.88 ± 0.14 ^b^	0.67 ± 0.23 ^c^	0.97 ± 0.16 ^ab^	0.85 ± 0.11 ^b^
TP(g/L)	42.55 ± 0.49 ^b^	42.92 ± 0.59 ^b^	44.27 ± 0.54 ^a^	45.88 ± 0.2 ^a^	42.89 ± 0.24 ^b^	42.97 ± 0.24 ^b^
ALB(g/L)	14.99 ± 0.2 ^b^	13.8 ± 0.29 ^b^	14.7 ± 0.23 ^b^	17.44 ± 0.17 ^a^	14.39 ± 0.12 ^b^	16.41 ± 0.18 ^a^
GLU(mmol/L)	5.54 ± 0.05	5.34 ± 0.02	5.73 ± 0.02	5.92 ± 0.04	5.43 ± 0.03	5.22 ± 0.08
TG(mmol/L)	3.49 ± 0.02 ^b^	2.96 ± 0.01 ^c^	3.85 ± 0.01 ^a^	3.99 ± 0.01 ^a^	3.52 ± 0.01 ^b^	3.17 ± 0.02 ^bc^
CHO(mmol/L)	4.82 ± 0.01	4.67 ± 0.01	4.58 ± 0.04	4.81 ± 0.03	4.64 ± 0.03	4.82 ± 0.04

Results are expressed as mean ± standard deviation (n = 3). Unique lowercase superscripts (a–c) denote significant differences among treatment groups (*p* < 0.05).

## Data Availability

The data can be available from the corresponding author upon reasonable request. The data are not publicly available due to privacy or ethical restrictions.
